# Quantitative Trait Loci Mapping and Marker Identification for Low Salinity Tolerance Trait in the Swimming Crab (*Portunus trituberculatus*)

**DOI:** 10.3389/fgene.2019.01193

**Published:** 2019-11-20

**Authors:** Jianjian Lv, Dongfang Sun, Deping Yan, Xingbin Ti, Ping Liu, Jian Li

**Affiliations:** ^1^Key Laboratory of Sustainable Development of Marine Fisheries, Ministry of Agriculture, Yellow Sea Fisheries Research Institute, Chinese Academy of Fishery Sciences, Qingdao, China; ^2^Function Laboratory for Marine Fisheries Science and Food Production Processes, Qingdao National Laboratory for Marine Science and Technology, Qingdao, China

**Keywords:** quantitative trait loci, low salinity tolerance, *Portunus trituberculatus*, association analysis, salinity marker

## Abstract

Low salinity is one of the most important abiotic factors that directly affect the abundance of the swimming crab, *Portunus trituberculatus*. Quantitative trait loci (QTL) mapping could be helpful in identifying the markers and genes involved in low salinity tolerance. In this study, two QTLs of low salt tolerance were mapped on linkage group 17 (LG17, 2.6–5.2 cM) based on a high-density linkage map. Ninety-five markers related to low salinity tolerance were identified *via* association analysis, and seventy-nine low salt-related candidate genes (including ammonium transport, aldehyde dehydrogenase, and glucosyltransferase) were screened from draft genome of the species *via* these markers. This represents the first report of QTL mapping for low salinity tolerance in the swimming crab, which may be useful to elucidate salinity adaptation mechanisms.

## Introduction

The swimming crab, *Portunus trituberculatus* (Crustacea: Decapoda: Brachyura), is an important marine species for fisheries and aquaculture. Its natural distribution is along the coastal waters of China, Korea, Japan, and other East Asian countries (Lv et al., 2013). Due to its fast growth, *P. trituberculatus* has become one of the most important economic species for marine aquaculture (Ren and Pan, 2014). In 2017, the total aquaculture yield of the crab reached 119,777 tons in China.

Salinity is one of the most important abiotic factors that directly affect the distribution and abundance of the swimming crab (Lv et al., 2013; Lv et al., 2015). During the breeding period, crabs are often exposed to low salinity stress due to rainstorms and water exchange, causing death and huge economic losses. Thus, in order to produce healthy crabs, it is necessary to breed varieties that are tolerant to low salinity. However, compared to growth, the heritability of salt tolerance traits is lower (Baoquan et al., 2010; Zheng et al., 2015). Compared with higher heritability traits, the genetic improvement of low heritability traits through routine selection is less efficient, but could be accelerated with the help of molecular marker assisted breeding (MAS) or molecular breeding technology (Poompuang and Hallerman, 1997; Martinez et al., 2007). To elucidate the potential genetic mechanisms underlying salt tolerance traits, some scholars tried to explore related genes by comparative transcriptome (Xu and Liu, 2011; Lv et al., 2013), and hundreds of potential salt tolerance related genes have been identified involved in crucial processes, such as ion transport processes, amino acid metabolism and synthesis processes, proteolysis process and chitin metabolic process. Among which, ion transport in gill was the research focus of elucidating the molecular mechanism of salinity adaptation (Freire et al., 2008; Romano and Zeng, 2012), and many ion transport related genes including Na^+^,K^+^-ATPase, V-type H^+^-ATPase and Na^+^,K^+^, 2Cl^−^ cotransporter were cloned and studied (Tsai and Lin, 2007; Garcon et al., 2011; Lv et al., 2016). However, the complex molecular mechanisms involved in salinity tolerance are still poorly understood, and there is a lack of valuable molecular markers for MAS.

An accurate high-resolution genetic linkage map is an essential tool for addressing genetics and genomics questions (Jiao et al., 2014; Yu et al., 2015). The development of such maps is also an important foundation for the genetic breeding of a species, and is indispensable for MAS (Andriantahina et al., 2013; Shao et al., 2015). With advances in sequencing technologies over the past 5 years, high-resolution linkage maps of several aquacultured crustaceans (e.g., *Penaeus monodon*, *Litopenaeus vannamei* and *E. sinensis*) have been constructed with thousands of markers identified (average marker distances < 1 cM) (Baranski et al., 2014; Cui et al., 2015; Yu et al., 2015). Recently, a high-density *P. trituberculatus* linkage map with 10,963 markers was mapped to 53 sex-averaged linkage groups, and had an average marker distance of 0.51 cM (Lv et al., 2017). Many important economic traits, including growth, disease resistance, and sex determination were mapped based on high-resolution linkage maps. However, there are no reports on quantitative trait loci (QTL) analyses for low salinity tolerance in crustaceans.

The aim of the present study was to identify QTL and markers for low salinity tolerance in the swimming crab. QTL mapping was performed based on a high-density linkage map constructed previously (Lv et al., 2017). Association analysis and verification among different populations were carried out to identify low salinity tolerance-related markers (salinity tolerant markers). As a result, QTLs for low salinity tolerance were mapped on LG17, and one salt-tolerance marker at the population level was identified. These results will be helpful to elucidate the mechanisms of low salinity tolerance in *P. trituberculatus*.

## Materials and Methods

### Data Collection

The materials and the data used in this study were the same as previously reported (Lv et al., 2017). Briefly, the QTL mapping population was an F1 full sib family containing 116 progenies derived from a female parent from the wild population of the Bohai Sea and a male parent from a F9 full sibling. Muscle tissues were sampled and immediately preserved in liquid nitrogen. Genomic DNAs were extracted using TIANamp Marine animal DNA extraction kit (TIANGEN, Beijing, China). The DNA concentration and integrity were evaluated *via* a NanoDrop 1,000 Spectrophotometer (NanoDrop, Wilmington, DE, USA) and electrophoresis in 1% agarose gel, respectively. Sequencing was performed on the Illumina HiSeq 2500 sequencing platform and markers were found *via* SLAF-seq (specific-locus amplified fragment sequencing). The SLAF library construction and sequencing follows what was previously described elsewhere (Lv et al., 2017). Finally, a high density linkage map with 10,963 markers and 0.51 cM marker interval was constructed.

### Quantitative Trait Loci Mapping

The salinity tolerance trait was collected for the full-sib family during low salt challenge process. In brief, the salinity content in the tank was gradually reduced to 5 ppt of salinity content by adding fresh water. This challenge experiment lasted for ∼72 h. Mortality was recorded every 3 h based on the appearance of dead crab (**Supplementary Table S1**). The method of QTL mapping was the same as in our previous work (Lv et al., 2017). Briefly, the multiple-QTL model mapping (MQM) method was using QTL analysis *via* MapQTL 4.0 software as described (Ooijen et al., 2000; Wei et al., 2014). 1 cM walking step was adopted in composite interval mapping (CIM). Approximately 95% confidence intervals were constructed using the two-LOD support rule (Ooijen, 1992). A minimum score of 3.0 likelihood-ratio statistic (LOD) was used to identify significant QTL in a particular genomic region, which was determined using 1,000 permutations. The phenotypic variance explained (*PVE*) was also calculated based on the mapping population variance *via* MapQTL4.0.

### Association Analysis

As a complementary approach to QTL mapping, the relationship between markers and salinity tolerance trait was further tested by association analysis using the same F1 family material as previously reported (Cui et al., 2015; Lv et al., 2018). ‘GWAF’ R package was used for association analysis in this work, which was designed mainly to analyze a batch of genotyped markers. The package is suitable for dichotomous or continuous phenotype, measured on subjects of families for genetic association (Chen and Yang, 2010). Logistic regressions *via* a generalized linear mixed effects model (GLMM) were used to test the genetic association between markers and phenotypes with the additive effect model. The functions ‘ lme.batch ‘ and ‘ gee.lgst.batch ‘ were implemented to perform a global test (*i.e.*, Wald χ 2 test) for genotype effects. Benjamini and Hochberg correction was applied to correct for Type I errors in multiple comparisons.

### Candidate Genes Identified

The markers located in the QTL interval or that showed significant association with the low salinity tolerance trait (p < 0.01) by association analysis (abbreviated as salinity marker), were aligned to the crab draft genome recently released (https://figshare.com/projects/De_novo_draft_genome_of_Portunus_trituberculatus_and_its_Hox_gene_cluster/61295). Then we retained the annotated scaffolds, which contained at least one known gene. These genes located in the scaffolds anchored by the salinity marker were considered candidate genes of low salinity tolerance trait. TBtools toolkit was used to carry out blast analysis between markers and genomic scaffolds (Chen et al., 2018), of which the E-value parameter was set to less than 1e-20.

### Validation of Salinity Markers

Firstly, the salinity markers were validated at the family level (36 individuals with the shortest survival time and 11 surviving individuals were selected from the QTL mapping family). Subsequently, to further conﬁrm the association at the population level, an additional challenge experiment was performed. Two hundred healthy crabs were selected from our research team’s core breeding population, which consisted of individuals derived from four wild geographical populations in 2005 (Li et al., 2013). The selected crabs were challenged in 5 ppt of salinity content as mentioned above. Based on the survival time, 20 individuals with the shortest and the longest survival time were selected, called sensitive group and tolerant group, respectively. Muscle tissues were sampled and immediately preserved in liquid nitrogen. Genomic DNAs were extracted using TIANamp Marine animal DNA extraction kit (TIANGEN, Beijing, China). The DNA concentration and integrity were evaluated *via* a NanoDrop 1000 Spectrophotometer (NanoDrop, Wilmington, DE, USA) and electrophoresis in 1% agarose gel, respectively. A total of ten markers with sufficient flanking regions (including four markers within the QTL interval and six salinity markers with p value of association analysis < 0.001), were selected to validate by PCR product sequencing (generally speaking, the smaller the p value, the greater the correlation with low salinity tolerance trait). All successfully amplified PCR target products of salinity markers were purified and sequenced with the automated sequencer ABI 3730 (Applied Bio-system). SNPs (single nucleotide polymorphism) were genotyped by sequencing chromatograms *via* Vector NTI 11.0 (Invitrogen). SNP site allele frequencies were calculated by SPSS 17.0 and compared by the Pearson chi-square test for the significance tests to confirm their association with the susceptibility/tolerance to salinity challenge. Results were considered statistically significant when *P* < 0.05.

## Results

### QTL Mapping of Low Salinity Tolerance Trait

**Figure 1:**
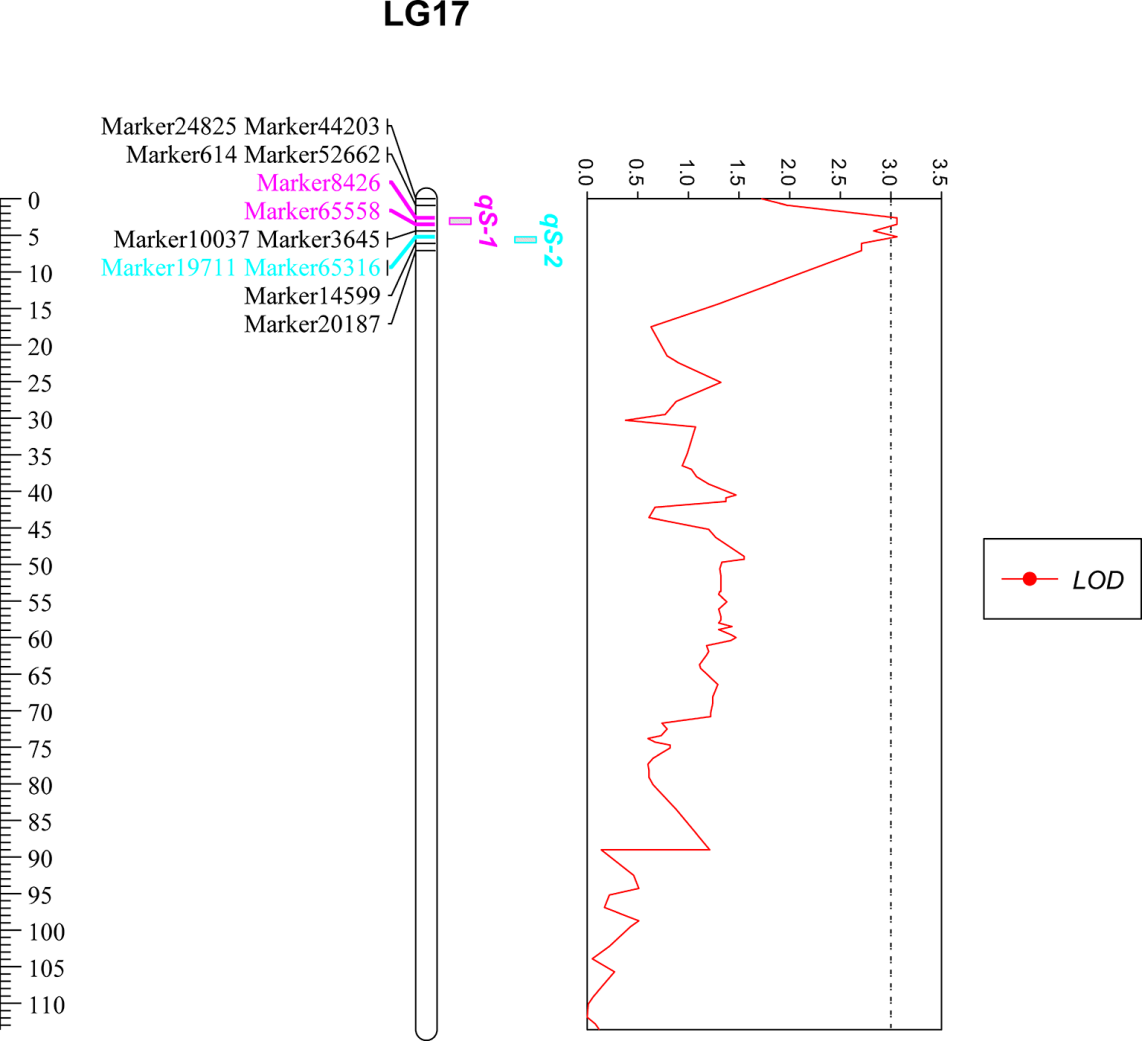
Quantitative trait loci mapping of low salinity tolerance trait in *P. trituberculatus*. The ruler shows the length of LG17, of which the unit is centimorgans (cM). Only markers near the QTL are displayed, among them, pink markers are located in the *qS-1* interval, blue markers are located in the *qS-2* interval. The four markers were overlapped with the salinity marker screening by association analysis. The red curve represents the LOD value of the markers on LG17.

On the basis of the high density linkage map (a total of 10,963 markers, with a mean marker interval of 0.51 cM), Two QTLs related to low salinity tolerance trait were detected by MapQTL 4.0 software, which were located on LG17 (2.6–5.2 cM) (**Table 1** and **Figure 1**). Each QTL interval contained two markers (a total of four markers were located in the two QTL intervals), and each marker contributed to *PVE* of 14.8% with LOD value of 3.06 (**Table 1**).

**Table 1:** Characteristics of low salinity tolerance trait related quantitative trait loci.

QTL	Linkage Group	Start (cM)	End (cM)	Marker Number	Marker ID	LOD	PVE
*qS-1*	17	2.624	3.494	2	Marker8426	3.06	14.8
					Marker65558	3.06	14.8
*qS-2*	17	5.233	5.233	2	Marker19711	3.06	14.8
					Marker65316	3.06	14.8

### Association Analysis

**Figure 2:**
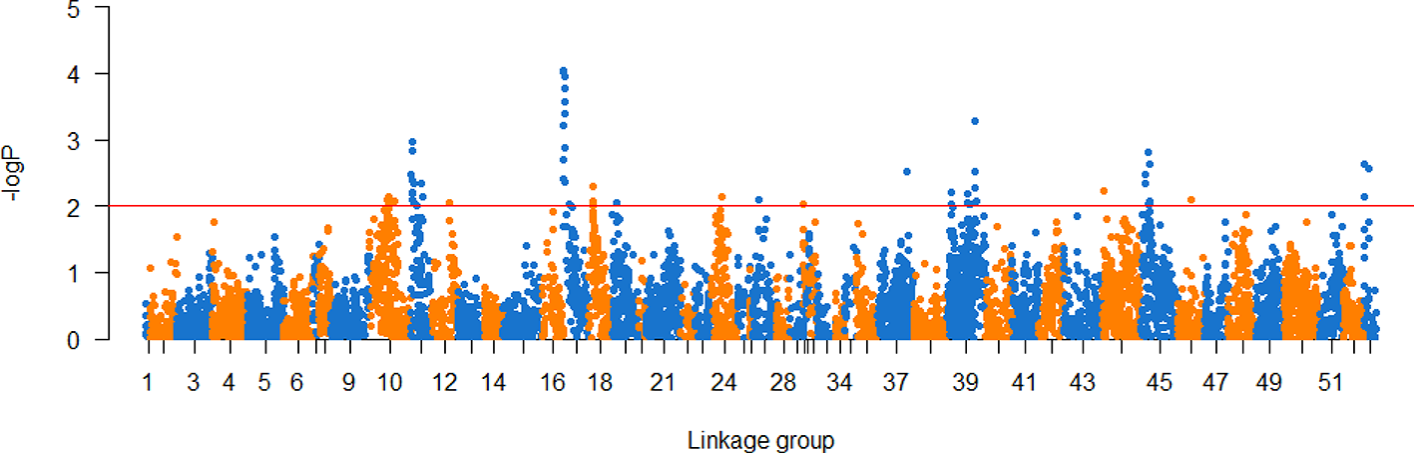
Association analysis of low salinity tolerance trait in *P. trituberculatus*.

The association analysis investigated a total of 36,976 markers in the mapping family, and 95 markers showed significant associations with low salinity tolerance trait (p < 0.01), among which, 21 markers with p value lower than 0.001 (**Supplementary Table S2**). Further analysis found that 50 of these markers were located on the linkage map, distributed on thirteen linkage groups (**Supplementary Table S3**) (**Figure 2**).

### Validation of Salinity Markers

To validate salinity markers, ten salinity markers (p < 0.001) with sufficient flanking regions sequenced were selected to sequence the PCR products of the parents of the QTL mapping populations (including four markers within the QTL interval). Finally, five of them could be sequenced with good quality (**Supplementary Table S4**), which were further genotyped in the QTL mapping population *via* PCR product sequencing. Chi-square test results showed that all five markers were significantly associated with low salinity tolerance traits (**Supplementary Table S5**). Subsequently, the five markers were validated in differentiated populations of low salinity tolerance traits, and found that one of the markers (marker8,426, p = 0.015) was significantly associated with low salinity tolerance trait at the population level (**Table 2**).

**Table 2:** Validation of salinity markers in population.

Locus	Genotype	Sensitive N (%)	Tolerant N (%)	X^2^ (P)
**Marker3645**	GG	9(0.45)	11(0.55)	0.420 (0.811)
	GA	7(0.35)	6(0.3)	
	AA	4(0.2)	3(0.15)	
**Marker10037**	TT	9(0.45)	7(0.35)	0.631 (0.729)
	TG	10(0.50)	11(0.55)	
	GG	1(0.05)	2(0.10)	
Marker8426*	TT	14(0.74)	7(0.35)	5.867 (0.015)
	TA	5(0.26)	13(0.65)	
	AA	0	0	
**Marker33515**	TT	10(0.50)	6(0.30)	2.450 (0.294)
	TC	1(0.05)	3(0.15)	
	CC	8(0.40)	11(0.55)	
**Marker19711**	CC	19(0.95)	20(1.00)	1.026 (0.311)
	GC	1(0.05)	0	
	GG	0	0	

*Chi-square test results showed that the marker were significantly associated with low salinity tolerance trait. (P < 0.05).

### Candidate Genes of Low Salinity Tolerance Trait

To detect the low salt tolerance-related genes, we compared 95 salinity markers sequences with all available genomic scaffolds *via* BLAT tool. The result showed that all markers could be aligned to genomic scaffolds, of which 25 markers were located in 23 annotated scaffolds. Further analysis found that there were 79 known genes in these scaffolds, including ammonium transport, aldehyde dehydrogenase (ALDH), glucosyltransferase (GT), *etc*. (**Supplementary Table S6**). The four markers located in the QTL interval anchored three scaffolds (scaffold1186, scaffold1442665 and scaffold1984813). A total of nine genes were identified from the scaffold1186 based on the annotation information, which contained two known genes, ammonium transporter and DNA mismatch repair protein (**Supplementary Table S7**).

## Discussion

Selection of low salinity tolerant *P. trituberculatus* is particularly important for aquaculture production. Thus, mapping QTLs and identifying markers linked to salinity tolerance are the first steps for improving stress tolerance in *P. trituberculatus* through MAS techniques. QTL mapping in *P. trituberculatus* has been performed for many traits that are economically important, including growth and sex, however, QTL mapping had not been reported for salinity tolerance yet.

Based on a high-density genetic linkage map (Lv et al., 2017), two QTLs of low salinity tolerance were mapped on LG17 (2.6–5.2 cM). Two markers within this QTL interval (Marker8,426 and Marker19,711) were screened again *via* association analysis and verified by further sequencing and genotyping in the QTL mapping population, which was helpful to confirm the accuracy of the QTL mapping in this work. We noticed that the two QTLs were close together, and the markers between the two QTLs (Marker10,037 and Marker3,645) were also verified to be associated with salt tolerance traits in the QTL mapping population. However, since the LOD value between the two QTLs is less than the threshold value, we still believe that the two QTLs cannot be merged into a single QTL in this study, which should be further validated using multiple families. To our knowledge, this is the first time that QTL analysis for low salinity tolerance has been performed on a high-density linkage map in crustaceans.

Compared with our previous work of growth QTL mapping (Lv et al., 2017), low salinity tolerance had fewer QTLs (12 *vs.* 2) and a lower *PVE* value (35.9 *vs.* 14.8%), which may be related to its lower heritability (0.53 *vs.* 0.18) (Baoquan et al., 2010; Zheng et al., 2015). In addition, salt tolerance are complex quantitative traits regulated by many genes (Jiang et al., 2019), its regulation mechanism may consist of multiple complex pathways when compared to growth traits. Previous research showed that 615 genes showed significant differential expression (DEG) during salinity stress, which were involved in many crucial processes including ion transport, amino acid metabolism and synthesis, proteolysis and chitin metabolic (Lv et al., 2013). The number of DEGs after low salinity stress (615) was significantly higher than what was found for the comparative transcriptome of different growth traits (117) (Lv et al., 2014). The results of association analysis in this work also support this hypothesis. A total of 95 salt-related markers were found on thirteen linkage groups, which suggests that salinity tolerance is a complex trait regulated by many genes distributed on different chromosomes. Because of the complex regulation mechanism of low salt tolerance, it is more difficult to accurately estimate the individual trait of salt tolerance, which may be another reason why there are fewer QTLs. This result also reminds us it would be more difficult to use MAS for genetic improvement for low salinity tolerance traits, and genome-wide selective breeding based on fine genome information may be a solution to the problem. However, the results of this study was based on the data from only one full sib family, and more families or populations should be investigated to facilitate a more complete mapping of salt tolerant QTLs in the future.

The combination of QTL mapping and association analysis can increase the range and accuracy of discovering association markers. In this work, the salinity markers found by QTL mapping and association analysis were partially overlapping, and mutual verification indicated the accuracy of the results. To further conﬁrm the association between salinity markers and resistance to low salinity, ten salinity markers (p < 0.001, including four overlapping markers), with sufficient flanking regions, were selected to validate across populations. Markers that could be successfully sequenced were all significantly associated with salt tolerance traits at the family level, among them marker 8,426 was significantly associated with low salinity tolerance trait at the population level (p = 0.015). In addition, we found that individuals with heterozygous genotype of marker 8,426 had better salinity tolerance than those with homozygous genotypes, and we will try to apply it in MAS for crabs.

We tried to blast these markers to the released genome data (Kim, 2019), and 79 genes were identified as candidate genes of low salinity tolerance. Previous studies on salinity tolerance of crustaceans mainly focused on osmoregulation in gill epithelial cells (McNamara and Faria, 2012; Romano and Zeng, 2012). A few genes related to ion transport in crustacean gills have been cloned (e.g., Na^+^/K^+^-ATPase, V-type H^+^-ATPase and Na^+^/K^+^/2Cl(^−^) cotransporter, *etc*.); however, our study found that such ion transport genes were not associated with low salinity tolerance. One possible reason is that these genes are not variable in this population. Another possibility is that ion transport-related genes play an important role in osmoregulation or salinity adaptation, but may not be key genes for salt tolerance traits.

Our previous results show amino acid content and concentration variation in muscle and hemolymph of *Portunus trituberculatus* at different salinities (Ping et al., 2017). Ammonium transport is a key process in amino acid metabolism (Song et al., 2011). Amino acid catabolism leads to the production of ammonium (NH4), which needs to be detoxified by ammonium transport (Cruz-Bustos et al., 2018). In this work, the ammonium transport gene was identified in the QTL interval *via* alignment of the salinity markers to the released genome database (https://figshare.com/projects/De_novo_draft_genome_of_Portunus_trituberculatus_and_its_Hox_gene_cluster/61295), which suggested the detoxify process regulated by ammonium transport was related to the salt tolerance trait.

Two genes, aldehyde dehydrogenase (ALDH) and glucosyltransferase (GT) were found on salinity markers, both of which involved in the process of ascorbate and aldarate metabolism of KEGG (Kyoto Encyclopedia of Genes and Genomes) pathway. ALDH belongs to a gene superfamily of NAD(P)^+^-dependent enzymes that catalyzes the irreversible oxidation of a wide range of endogenous and exogenous aromatic and aliphatic aldehydes (Chen et al., 2014). ALDH has been systematically investigated in several plant species, including *Arabidopsis* and tobacco (Li et al., 2003; Sunkar et al., 2003). Recent studies suggest that ALDH can provide protection from salinity stress by generating osmoprotectants, such as glycine betaine (Zarei et al., 2018). GT is important in the endoplasmic reticulum for protein quality control, which plays an important role in plant vegetative development, and impairs the response to several forms of abiotic and biotic stress (Ritter and Helenius, 2000). GT mutant plants are more sensitive than wild type plants when grown under salinity stress, exhibiting a significant decrease in fresh weight (Blanco-Herrera et al., 2015). However, few studies have focused on these genes in studies of animal salinity tolerance, and will be the focus of our future research.

## Conclusions

The swimming crab is an important marine species for fisheries and aquaculture. To elucidate the potential genetic mechanisms underlying economic traits, related genes were explored by comparative transcriptome and QTLs of important economic traits (including growth and sex determination) were mapped. However, there are no reports on QTL analyses for low salinity tolerance of this crab. In this work, based on a high-density linkage map, QTLs of low salinity tolerance in *P. trituberculatus* were mapped on LG17 for the first time. Five low salinity-related markers were found at the family level, including one marker which was closely related to low salinity tolerance at the population level. Low salinity-related genes, including ammonium transport, aldehyde dehydrogenase, and glucosyltransferase were identified. Our results are helpful in elucidating the mechanism of low salinity tolerance, and may help accelerate MAS in crabs.

## Data Availability Statement

The raw data supporting the conclusions of this manuscript will be made available by the authors, without undue reservation, to any qualified researcher.

## Ethics Statement

This study was carried out in accordance with the recommendations of ‘the Yellow Sea Fisheries Research Institute’. The protocol was approved by ‘the Yellow Sea Fisheries Research Institute’. The crabs used in the present study were marine-cultured animals, and all of the experiments were conducted according to the regulations of the local and central government. Prior to the sampling, all crabs will be treated with cold shock method to minimize suﬀering.

## Author Contributions

JLi and PL conceived and supervised the project. JLv and DS supplied the experimental animals. JLv contributed to the QTL mapping and association analysis. DY and XT contributed to the validation of salinity markers. JLv wrote the manuscript. All authors read and approved the final manuscript.

## Funding

This research was supported by the National Key R&D Program of China (2018YFD0900303-03), National Natural Science Foundation of China (41776160 and 41576147), Shandong Province Key Development Program for Research (2018GSF121030), Qingdao Applied Basic Research Project (17-1-1-95-jch), Efficient Eco Agriculture Innovation Project of Taishan Leading Talent Project (No. LJNY2015002), and Special Scientific Research Funds for Central Non-profit Institutes, Yellow Sea Fisheries Research Institute (Project 20603022018027).

## Conflict of Interest

The authors declare that the research was conducted in the absence of any commercial or financial relationships that could be construed as a potential conflict of interest.
